# NORMATIVE VALUES IN HEALTHY ADULTS FOR THE 6-MINUTE AND 2-MINUTE WALK TESTS IN BELGIUM AND VIETNAM: IMPLICATIONS FOR CLINICAL PRACTICE

**DOI:** 10.2340/jrm.v56.18628

**Published:** 2024-03-19

**Authors:** Duy Thanh NGUYEN, Massimo PENTA, Claire QUESTIENNE, Johanne GARBUSINSKI, Nguyen Van CHINH, Chloé SAUVAGE

**Affiliations:** 1 Faculty of Nursing and Medical Technology, University of Medicine and Pharmacy at Ho Chi Minh City, Vietnam; 2 Faculty of Motricity Sciences, Université Libre de Bruxelles, Belgium; 3 Institute of NeuroScience, Université catholique de Louvain, Louvain-la-Neuve, Belgium; 4 Arsalis, Glabais, Belgium; 5 Department of Neurorehabilitation, Erasme Hospital, Université Libre de Bruxelles, Belgium

**Keywords:** reference values, rehabilitation, walk test, healthy subjects, geographic variability

## Abstract

**Objective:**

To establish reference values for the 6-minute walk test (6MWT) and 2-minute walk test (2MWT) distances, to investigate the correlation between these 2 tests, and to establish prediction equations for these distances in healthy populations of Belgium and Vietnam.

**Design:**

Cross-sectional study.

**Subjects and methods:**

The 6MWT and 2MWT were administered to a convenience sample of 239 Belgian and 303 Vietnamese participants between the ages of 18 and 80 years.

**Results:**

The mean (standard deviation; SD) 2MWT distances were 215 (32.8) m for Belgian participants and 156 (25.5) m for Vietnamese participants. The mean (SD) 6MWT distances were 625 (90.7) m for Belgian participants and 449 (70.4) m for Vietnamese participants. The Pearson correlation coefficient between the 2 tests was 0.901 (*p* < 0.001) for Belgian participants and 0.871 (*p* < 0.001) for Vietnamese participants. Age and sex were the 2 most important predictors of walking distance, followed by body mass index for Belgium and height for Vietnam. The adjusted R² ranged from 0.31 to 0.49 across 4 predictive equations.

**Conclusion:**

These results can be used to determine the presence of walking performance deficits and to guide future studies. The 2MWT is suggested as a useful and convenient alternative to the 6MWT for assessing walking performance in clinical practice.

LAY ABSTRACT

This study aimed to establish reference values, correlations and prediction equations for the 6-minute walk test and 2-minute walk test in healthy individuals from Belgium and Vietnam. A total of 239 Belgian and 303 Vietnamese volunteers, age range 18–80 years, participated in this cross-sectional study. Results showed that there were differences in the walking distances between the 2 countries, with Belgian participants walking greater distances than Vietnamese participants in both the 2-minute and 6-minute walk tests. A strong correlation was observed between 6-minute walk test and 2-minute walk test distances for both groups. Age and sex were the 2 most important predictors of walking distance, along with body mass index for Belgium and height for Vietnam. These findings provide clinicians with age- and sex-specific reference values to assess walking performance and guide future studies. The 2-minute walk test is suggested as a convenient alternative to the 6-minute walk test for assessing walking performance in clinical practice.

Timed walking tests are safe, simple, and inexpensive tools used to evaluate functional exercise capacity (1). The 6-minute walk test (6MWT) is widely used to measure functional exercise performance, response to treatment and disease progression across a wide range of respiratory and neuromuscular diseases in clinical settings (2, 3). However, the 6MWT is time-consuming and often not applicable in patients who are hospitalized with an acute stage of disease or who have severe impairment of the lower limbs (4). Previous studies have found that the 2-minute walk test (2MWT) is highly related to the 6MWT and may be an appropriate alternative when assessing walking capability in patients with multiple sclerosis (5), stroke (6), amputation (7), and a wide range of neuromuscular diseases (4).

The optimal clinical use of the 6MWT and the 2MWT requires normative reference values for clinicians to interpret the walking performance of patients relative to their age and sex (8). Normative reference values involve mean performance data or prediction equations to assess any deviation from the values for healthy individuals (8). There are studies reporting normative reference values for either 6MWT or 2MWT in healthy people from countries in America (9, 10), Europe (11, 12), Asia (13–18), Australia (19, 20), and Africa (21). These studies have provided age- and sex-specific reference values for 6MWT and 2MWT in various countries. However, there are substantial differences in the norms for the distances covered in the 6MWT and 2MWT in the same age groups across different countries or regions. In addition, it has been shown that normative values established for one region may be inappropriate for another region, as multinational studies have indicated their impracticability for broader use (22, 23). Prediction equations established for 6MWT and 2MWT distances (9, 10, 17, 24), also offer a region-specific assessment of the determinants of walking performance that can further increase the accuracy of clinical assessment, as they differ substantially between studies and may not be appropriate for different regions.

This study targets the general adult population of Belgium and Vietnam with the aim of developing normative values for the 2MWT and 6MWT for both countries. In the long run, the lack of reference values and prediction equations for the 6MWT and 2MWT specific to healthy participants in Belgium and Vietnam prevents accurate patient assessments in clinical settings in these countries. Therefore, the objectives of the current study were: (*i*) to establish reference values for the 6MWTand 2MWT distances in healthy Belgian and Vietnamese adults aged 18–80 years; (*ii*) to investigate the correlation between 6MWT and 2MWT distances of the participants in these 2 countries; and (*iii*) to establish prediction equations to predict 6MWT and 2MWT distances in healthy Belgian and Vietnamese populations.

## METHODS

### Participants

A convenience sample of healthy adults from Belgium and Vietnam participated in the study. Participants were healthy volunteers who were able to follow test instructions, were at least 18 years old, had no history of neuromuscular, musculoskeletal, or cardiopulmonary disease that limited their walking ability, and did not use a walking aid. Participants were medical professionals and workers from local hospitals or other local activities, students and teachers at local universities, farmers, and volunteers in the community. The study protocol was approved by the Ethics Committee of the Erasme Hospital (Belgium) and the Human Research Ethics Committee of University of Medicine and Pharmacy at Ho Chi Minh City (Vietnam).

### Procedure

Participants were instructed to wear comfortable clothing, usual footwear and avoid doing vigorous exercises or eating heavy meals within 2 h before walking tests. All participants had to sit near the starting point and rest for 15 min. Data obtained included demographic characteristics, such as sex, age, height, weight, body mass index (BMI) and physical activity level, using the International Physical Activity Questionnaire short form (IPAQ-SF) prior to walking tests. The order of the 2MWT and 6MWT was assigned randomly, and a 15-min rest was imposed between both tests.

### Six-minute and 2-minute walk tests

The 6MWT was administered according to the American Thoracic Society (ATS) guidelines (25) and the 2MWT was a revised version of the 6MWT (26). Participants were instructed to walk as far as they could and were allowed to slow down or stop and continue to walk again after they recovered during the test period. After 1 min had elapsed, the evaluator provided standard encouragement with an even tone, such as “You are doing well; you have 5-, 4-, 3-, 2-, or 1-min left”. The subject stopped at 2 min or 6 min, and the distance covered was measured to the nearest metre. The 2MWT and 6MWT were performed over a flat 30-m course. Although it is recommended to conduct multiple trials (27), all participants performed both walk tests without any prior trial, in order to provide practical values for comparison with results from daily clinical practice where patients are often tested only once (11).

### Data analysis

Demographic characteristics were compared between both countries using a Mann–Whitney *U* test or χ^2^ test. Differences in walked distance for the 2MWT and 6MWT were tested with a 3-way analysis of variance (ANOVA) as a function of sex, country, and age group (18–29, 30–39, 40–49, 50–59, 60–69 and 70–80 years age groups). A Tukey post-hoc test was used to determine different subject factor values, where significant effects were observed.

Pearson’s correlation coefficients were computed between 2MWT and/or 6MWT distances and demographic variables in each country. The strength of the relationships between variables were estimated according to the correlation coefficient value (absent or weak relationship, *r* < 0.25; weak to moderate, 0.25 ≤ *r* < 0.5; moderate to strong, 0.5 ≤ *r* < 0.75; or strong relationship, *r* ≥ 0.75) (28).

Prediction equations for distance walked and predictive role of clinical variables (i.e. sex, age, height, weight, BMI, and IPAQ-SF) were obtained from stepwise multiple linear regressions. The analysis allowed the best predictors of walked distance (2MWT and 6MWT) to be identified in each country and only the statistically significant predictors were kept in the final model for each country. The level of significance for all statistical tests was set to 0.05. All analyses were performed using the R language for statistical computing (version 4.0.5; R Foundation for Statistical Computing, Vienna, Austria).

## RESULTS

There were 239 Belgian and 303 Vietnamese participants assessed in the study. Participants’ characteristics are shown in Table I. Overall, the Belgian participants had significantly higher values in height, weight, BMI and physical activity level than the Vietnamese participants, although there was a significantly higher proportion of females in Vietnam in the current sample.

**Table I:** Demographic data for Belgium and Vietnamese subjects (N = 542)

Characteristic	Belgium (*n* = 239)	Vietnam (*n* = 303)	*p*-value
Sex (female/male), *n*	120/119	184/119	0.018
Age, years median (IQR)	44.0 (30.0–57.5)	47.0 (30.0–61.0)	0.032
Height, cm, median (IQR)	171.0 (165.0–178.0)	158.0 (152.5–165.0)	< 0.001
Weight, kg, median (IQR)	72.0 (64.0–84.0)	56.0 (50.0–65.0)	< 0.001
Body mass index, kg/m^2^, median (IQR)	24.3 (22.0–27.4)	22.8 (20.4–24.8)	< 0.001
IPAQ-SF, MET-min/week, median (IQR)	2,772 (1400–4718)	1,176 (198–3439)	< 0.001

IQR: interquartile range; IPAQ-SF: International Physical Activity Questionnaire Short Form.

The mean (SD) of the 2MWT distances were 215 (32.8) m and 156 (25.5) m for Belgian and Vietnamese participants, and the mean (SD) 6MWT distances were 625 (90.7) m and 449 (70.4) m for Belgian and Vietnamese participants, respectively. The 3-way ANOVA showed a significant difference between countries for distances walked during the 2MWT (mean difference 59.1 m, *p* < 0.001) and the 6MWT (mean difference 175.8 m, *p* < 0.001). Sex also showed a significant effect, with males walking higher distances than females in both countries for the 2MWT (mean difference 21.9 m, *p* < 0.001) and for the 6MWT (mean difference of 63.6 m, *p* < 0.001). A decrease in distance walked with age was also observed whatever the sex in both countries, showing that participants younger than 29 years walked a greater distance (204 m for 2MWT and 583 m for 6MWT) than participants between 30 and 59 years of age (184 m for 2MWT and 533 m for 6MWT) and participants over 60 years of age (158 m for 2MWT and 527 m for 6MWT). No significant interaction was observed between factors either for the 2MWT or for the 6MWT, indicating that sex, age group and country are factors that independently affect walking performance. The normative reference values of distance walked in 2MWT and 6MWT of the Belgian and Vietnamese participants are shown in Table II.

**Table II:** Normative reference values for 2-minute walk test (2MWT) and 6-minute walk test (6MWT) distances for Belgium and Vietnamese subjects

	Belgium (N = 239)			Vietnam (N = 303)		
	*n*	2MWT (m) Mean (95% CI)	6MWT (m) Mean (95% CI)	*n*	2MWT (m) Mean (95% CI)	6MWT (m) Mean (95% CI)
Women						
18–29 years	30	220 (213–227)	642 (624–660)	44	178 (172–184)	507 (492–522)
30–39 years	20	207 (197–217)	599 (571–627)	26	153 (147–159)	444 (429–459)
40–49 years	21	208 (196–220)	604 (570–638)	27	147 (142–152)	428 (409–447)
50–59 years	24	203 (193–213)	595 (565–625)	32	146 (142–150)	423 (410–436)
60–69 years	12	201 (178–224)	593 (537–649)	36	139 (133–145)	397 (379–415)
70–80 years	13	187 (168–206)	530 (484–576)	19	114 (106–122)	327 (302–352)
Men						
18–29 years	29	247 (236–258)	700 (673–727)	27	182 (173–191)	518 (496–540)
30–39 years	21	233 (220–246)	667 (637–697)	23	171 (163–179)	495 (472–518)
40–49 years	23	224 (213–235)	655 (620–690)	21	168 (158–178)	482 (458–506)
50–59 years	21	214 (204–224)	628 (597–659)	21	162 (156–168)	466 (449–483)
60–69 years	12	202 (175–229)	608 (525–691)	19	146 (138–154)	431 (405–457)
70–80 years	13	192 (157–227)	563 (462–663)	8	141 (128–154)	403 (371–435)

95% CI: 95% confidence interval.

**Fig. 1:**
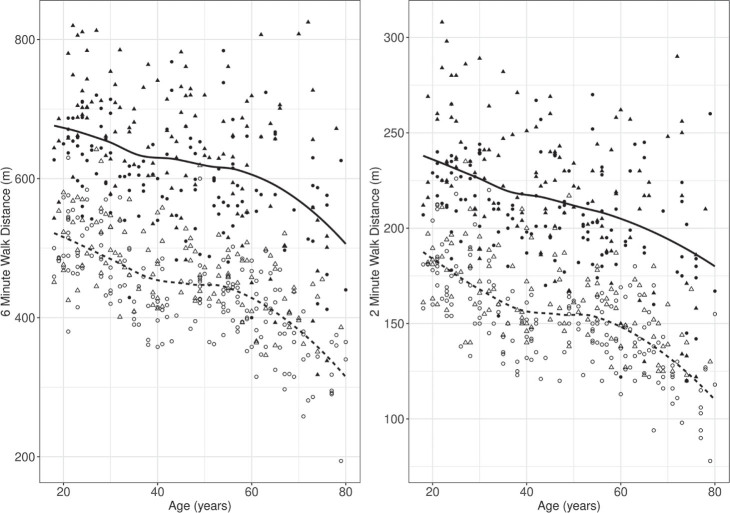
Evolution of 2-minute walk test (2MWT) and 6-minute walk test (6MWT) distances as a function of age in Belgium and Vietnam (*dashed line:* Vietnamese subjects; *black triangle:* Belgian male; *black circle:* Belgian female; *white triangle:* Vietnamese male; *white circle:* Vietnamese female).

The relationships between age and the 2MWT and 6MWT distances of all participants in 2 countries are shown in Fig. 1.

The Pearson correlation coefficient between 6MWT and 2MWT distances was 0.901 (*p* < 0.001) for Belgian participants and 0.871 (*p* < 0.001) for Vietnamese participants. Correlations between independent variables and 2MWT or 6MWT distances are shown in Table III. There were weak to moderate significant correlations between 2MWT and 6MWT distances with age, height, sex, and BMI for Belgian participants. In Vietnam, age demonstrated a strong significant correlation with distance walked, followed by height and sex, which showed a weak to moderate significant correlation. BMI and weight showed weak, though significant, correlation with walking distance for Vietnamese participants.

**Table III:** Correlation between independent variables and the 2-minute walk test (2MWT) and 6-minute walk test (6MWT) distances

Independent variables	Belgium (*n* = 239)		Vietnam (*n* = 303)	
	2MWT	6MWT	2MWT	6MWT
Age	–0.421 (< 0.001)	–0.390 (< 0.001)	–0.637 (< 0.001)	–0.630 (< 0.001)
Height	0.297 (< 0.001)	0.306 (< 0.001)	0.439 (< 0.001)	0.460 (< 0.001)
Sex	–0.255 (< 0.001)	–0.259 (< 0.001)	–0.291 (< 0.001)	–0.309 (< 0.001)
BMI	–0.281 (< 0.001)	–0.286 (< 0.001)	–0.147 (0.030)	–0.136 (0.052)
Weight	–0.077 (0.233)	–0.075 (0.250)	0.141 (0.014)	0.163 (0.004)
IPAQ-SF	–0.011 (0.864)	–0.045 (0.492)	0.029 (0.643)	0.041 (0.502)

IPAQ-SF: International Physical Activity Questionnaire Short Form.

Correlation analysis showed that participants in both countries walked shorter distances if they were older, shorter, female, and presented a higher BMI. Prediction equations for each walking distance were derived for each country. Stepwise linear regression showed that the 2 most important predictors of walking distance were age and sex then, depending on the country, either BMI for Belgium or height for Vietnam. Detailed regression equations are shown in Table IV. The adjusted R-squared values derived from successive stepwise models of 6MWT and 2MWT distances in both countries are as follows: for the Belgian 6MWT prediction equation, it was shown that the R-squared value increased from 0.15 (age only) to 0.21 (age and sex), and to 0.30 (age, sex, and BMI); for the Belgian 2MWT, the R-squared increased from 0.18 (age only) to 0.24 (age and sex), and then to 0.32 (age, sex, and BMI); for the Vietnamese participants 6MWT prediction equation, the R-squared value increased from 0.39 (age only) to 0.47 (age and sex), and to 0.49 (age, sex, and BMI); for the Vietnamese participants 2MWT the R-squared value increased from 0.40 (age only) to 0.47 (age and sex), and to 0.49 (age, sex, and height).

**Table IV:** Multiple linear regression model for 2-minute walk test (2MWT) and 6-minute walk test (6MWT) distances in Belgium and Vietnam

Variables	Belgium				Vietnam			
	2MWT		6MWT		2MWT		6MWT	
	B	SE	B	SE	B	SE	B	SE
Constant	317.00		905,38		106.00		276.60	
Age, years	–0.73	0.10	–1,83	0.29	–0.85	0.06	–2.28	0.18
Sex	–20.44	3.60	–57,85	10.08	–6.86	3.03	–19.34	8.34
BMI, kg/m^2^	–2.34	0.44	–6,78	1.24	NA	NA	NA	NA
Height, cm	NA	NA	NA	NA	0.59	0.19	1.82	0.53
Belgium								
2MWT distance = 317.0 – (0.73 × age) – (20.44 × sex) – (2.34 × BMI) R^2^ = 0.31								
6MWT distance = 905.38 – (1.83 × age) – (57.85 × sex) – (6.78 × BMI) R^2^ = 0.30								
Vietnam								
2MWT distance = 106.0 – (0.85 × age) + (0.59 ×height) – (6.86 × sex) R^2^ = 0.48								
6MWT distance = 276.60 – (2.28 × age) + (1.82 × height) – (19.34 × sex) R^2^ = 0.49								

Sex is categorized as female = 1 and male = 0. B: unstandardized coefficients; SE: standard error; NA: not applicable; BMI: body mass index.

## DISCUSSION

This study established reference values for the 6MWT and 2MWT in healthy individuals aged 18–80 years in Belgium and Vietnam. There were strong correlations between the 6MWT and 2MWT performance in both countries. The study also developed predictive equations for the distance walked in both tests, based on age and sex for both countries together, with either BMI for Belgium or height for Vietnam. These findings provide valuable information for clinicians and researchers in assessing locomotor performance with walking tests.

This study provides normative reference values for the 6MWT and 2MWT in healthy people from Belgium and Vietnam. The normative values are presented by sex and age categories, aligning with previous studies on walking performance (10, 12, 14, 16) and the sample size of the study was adequate for generating normative values by age and sex categories (8, 29). In agreement with the literature, there was a decrease of 6MWT and 2MWT distance with age, and men walked further than women (10, 23). Belgian participants had similar performances to participants from Portugal (12), Norway (11), and Australia (19) in the 6MWT, while Vietnamese participants had similar performances to participants from Saudi Arabia (14, 30), but not as high as those from Singapore (22). In the 2MWT, Belgian participants covered a similar distance to that of Brazilian participants (9), but a longer distance than USA participants (10), while Vietnamese participants covered a shorter distance than Chinese participants (17). A similar relationship to age and sex on the performance to the 6MWT and 2MWT in the current study compared with previous studies supports the validity of the reported age-and sex-specific normative values.

The distance covered by Belgian participants in both tests is significantly greater than that of Vietnamese participants in the corresponding age and sex group. This discrepancy in the 6MWT distance is observed not only between Belgian and Vietnamese participants, but also between different ethnic groups and countries. Previous research has reported that healthy white Americans walk a mean of 40 m greater distance in the 6MWT than their African-American counterparts after correcting for age, sex, height and weight (2). Differences in the walking distance also exist among Asians compared with Caucasians owing to the shorter stature of Asians and their higher percentage of body fat for an equivalent BMI (31). The differences in performance in walking tests can be explained by a complex set of factors, including demographic and anthropometric characteristics, cultural factor, habitual walking patterns, and lifestyle aspects (8). Moreover, the mood, attitude, and motivation of the subject, as well as psychological factors, may also play an essential role in the distance walked during the test (23).

The results of the current study show a strong correlation between the 2MWT and 6MWT in healthy Belgian (0.901) and Vietnamese (0.871) participants. This is the first study to investigate this correlation in healthy adults. Previous study only reported the correlation calculated by distance covered in the first 2 min of the 6MWT with the same whole 6MWT (0.968) of the healthy participants (32). However, this was based on only 1 walking trial, which led participants to maintain a consistent walking pace throughout the trial. In contrast, the current study included 2 separate walking trials, which may have contributed to the lower, but still strong, correlation between the 2 tests. Previous studies have also shown high correlations between these 2 tests in people with different health conditions, including those with neuromuscular diseases (0.99) (4), amputations (0.95) (7), multiple sclerosis (0.947) (5), and stroke (0.997) (6). Taken together, these findings suggest that the 2MWT may be a feasible substitute for the 6MWT in clinical practice, for both healthy people and for patients with severe pathologies, as it has the potential to reduce participant fatigue, save time and better target highly disabled patients in clinical settings.

The results of this study indicate that the equations predicting the 6MWT and 2MWT distance for Belgium and Vietnam have R-squared values that range from 0.3 to 0.49, which is similar to the range of equations reported previously (0.04–0.78, median = 0.46) (8). The R-squared value of age as an independent variable makes the largest contribution to the variability in the 6MWT and 2MWT reference values, ranging from 0.15 to 0.40. Other variables contribute only 2–8% of the variance in walked distance. The results also suggest that Vietnamese participants are more sensitive to age-related changes in locomotor performance than Belgian participants.

The equations in the current study share 2 commonly used predictors: age and sex, which can be determined rapidly through self-report and observation. In fact, age and sex are present in most prediction equations for 6MWT and 2MWT from different countries (8). The age variable is included as variable predictor in almost all predicting equations, except in a study with a narrow age range focused on young populations (17).

The prediction equations for both countries differ in their additional predictors. The Belgian equation includes BMI, similar to the equation for Caucasian participants (10, 20), while the Vietnamese equation includes including height, which is consistent with previous observations in Asia (17, 30). The BMI predictor appeared only in the Asian equation (33) that specifically analysed obese participants. Therefore, in addition to the common factors of age and sex, researchers should consider additional independent variables, such as BMI or height, based on geographical and anthropometric factors.

Despite exceeding the American College of Sport Medicine’s recommended recommended physical activity level (34), our sample’s median physical activity falls below that of the general population in both Belgium (35) and Vietnam (36). In addition, correlations between walking performance and physical activity were either absent or weak (Table III). Therefore, physical activity does not influence walking performance in the current study in a different way from in the general population of Belgium and Vietnam.

The current study had some limitations related to sample size, with the elderly group being slightly underrepresented. Participants of 60 years or more reported a slightly higher spread in walking performance (SD 42.4 m in 2MWT and 126.0 m in 6MWT) than participants younger than 60 years of age (SD 37.6 m in 2MWT and 107.1 m in 6MWT). In addition, differences in sex distribution were observed between both countries, with a higher proportion of females in Vietnam. These factors may have affected the study’s findings. The reference values reported in this study were based on unrepeated trials, which may have resulted in lower walking performance than if based on the maximum performance across multiple trials according to the ATS guidelines (25). However, these reference values reflect the same performance as achieved in clinical practice with a single trial per patient, and may serve as a useful benchmark for setting achievable treatment goals (8).

In conclusion, this study provides age- and sex-specific reference values and equations for the 6MWT and 2MWT for a broad age range and a representative sample size in healthy individuals from Belgium and Vietnam. The findings can be used to determine the presence of walking performance deficits, establish rehabilitation goals and guide future research. The 2MWT is suggested as a useful and convenient alternative to the 6MWT for assessing walking performance in clinical practice.
